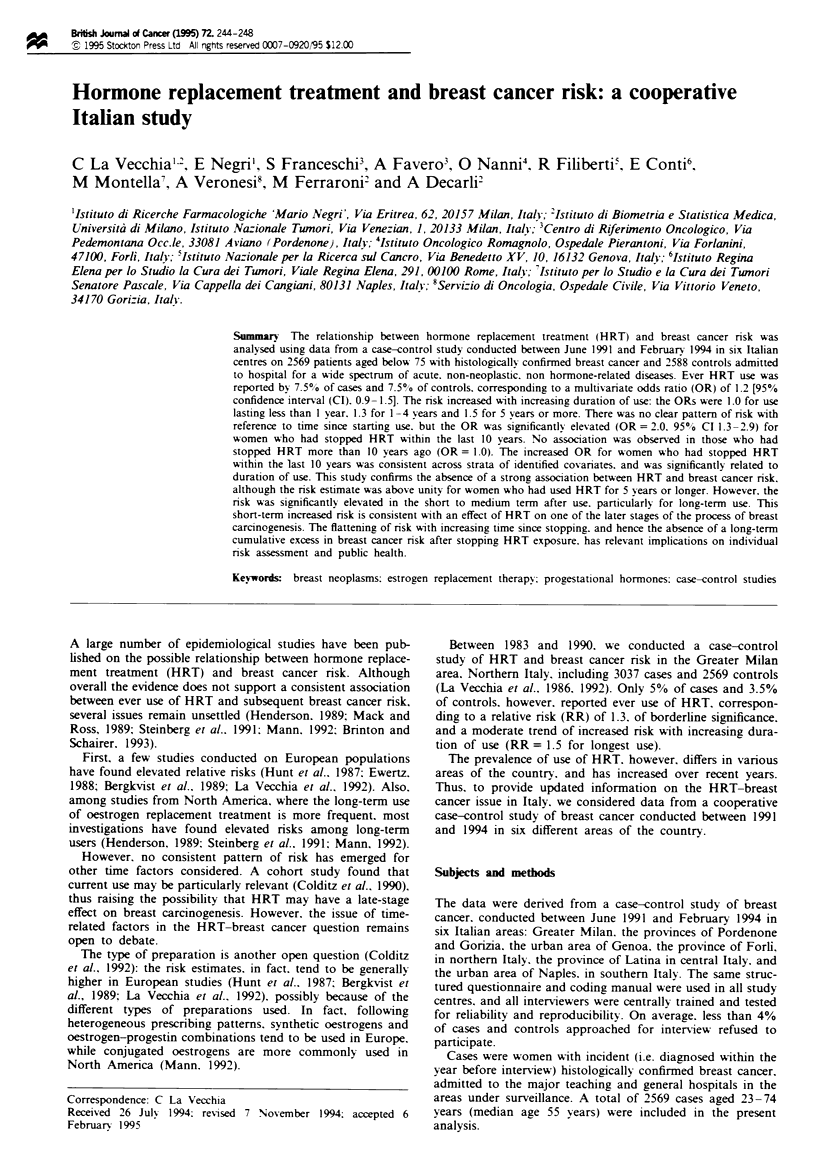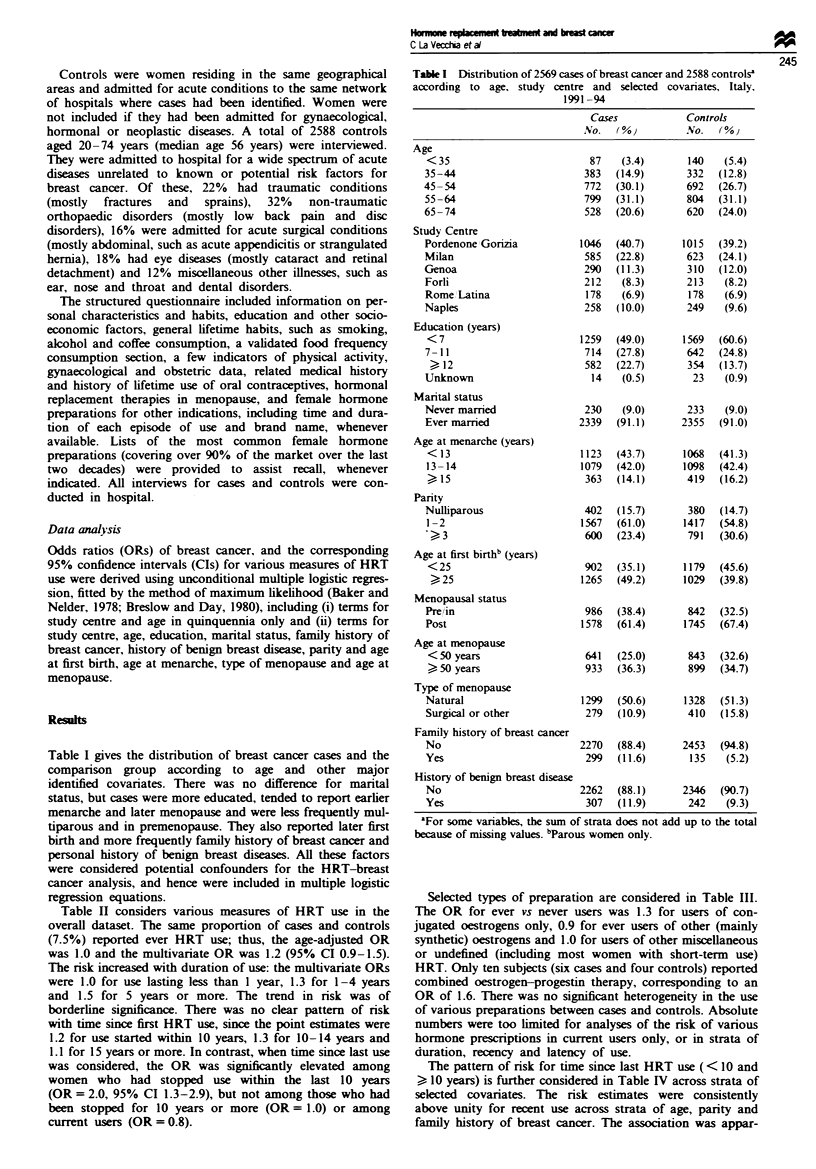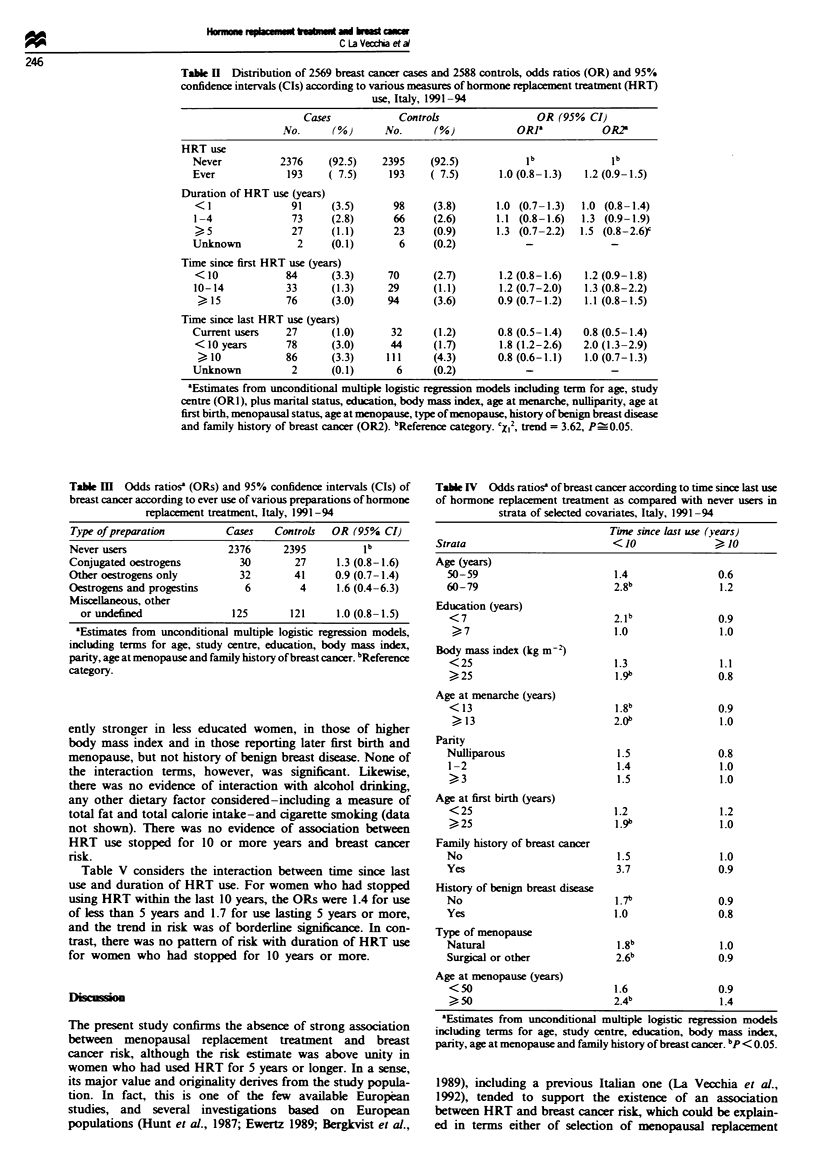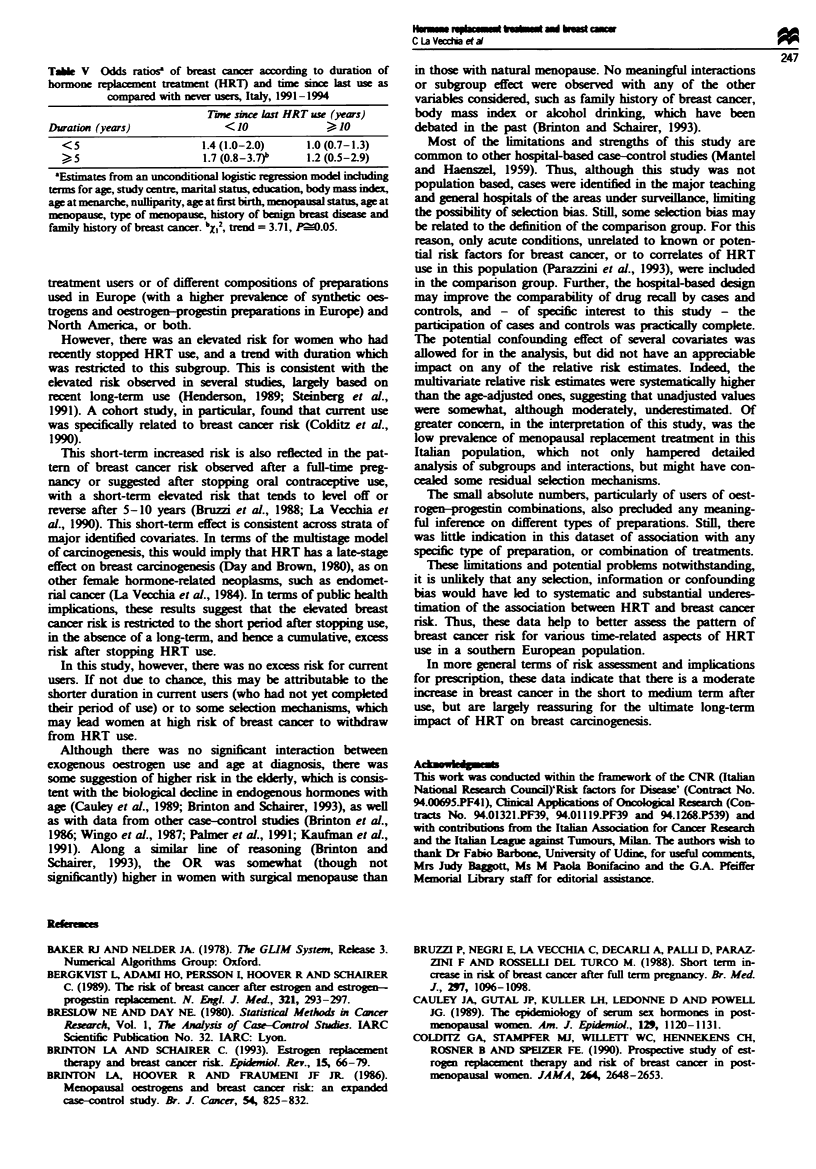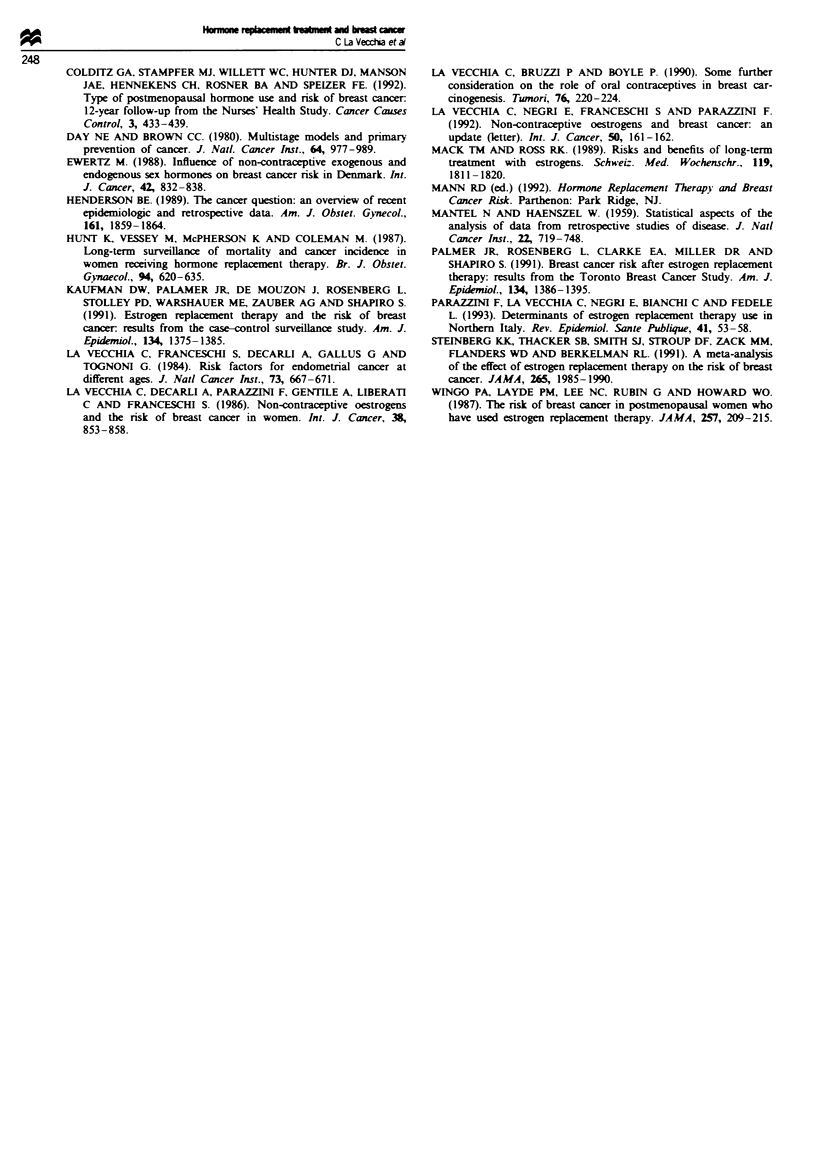# Hormone replacement treatment and breast cancer risk: a cooperative Italian study.

**DOI:** 10.1038/bjc.1995.310

**Published:** 1995-07

**Authors:** C. La Vecchia, E. Negri, S. Franceschi, A. Favero, O. Nanni, R. Filiberti, E. Conti, M. Montella, A. Veronesi, M. Ferraroni

**Affiliations:** Istituto di Ricerche Farmacologiche Mario Negri, Via Eritrea, Milan, Italy.

## Abstract

The relationship between hormone replacement treatment (HRT) and breast cancer risk was analysed using data from a case-control study conducted between June 1991 and February 1994 in six Italian centres on 2569 patients aged below 75 with histologically confirmed breast cancer and 2588 controls admitted to hospital for a wide spectrum of acute, non-neoplastic, non hormone-related diseases. Ever HRT use was reported by 7.5% of cases and 7.5% of controls, corresponding to a multivariate odds ratio (OR) of 1.2 [95% confidence interval (CI), 0.9-1.5]. The risk increased with increasing duration of use: the ORs were 1.0 for use lasting less than 1 year, 1.3 for 1-4 years and 1.5 for 5 years or more. There was no clear pattern of risk with reference to time since starting use, but the OR was significantly elevated (OR = 2.0, 95% CI 1.3-2.9) for women who had stopped HRT within the last 10 years. No association was observed in those who had stopped HRT more than 10 years ago (OR = 1.0). The increased OR for women who had stopped HRT within the last 10 years was consistent across strata of identified covariates, and was significantly related to duration of use. This study confirms the absence of a strong association between HRT and breast cancer risk, although the risk estimate was above unity for women who had used HRT for 5 years or longer. However, the risk was significantly elevated in the short to medium term after use, particularly for long-term use. This short-term increased risk is consistent with an effect of HRT on one of the later stages of the process of breast carcinogenesis. The flattening of risk with increasing time since stopping, and hence the absence of a long-term cumulative excess in breast cancer risk after stopping HRT exposure, has relevant implications on individual risk assessment and public health.


British Journal of Cancer (1995) 72, 244-248

© 1995 Stockton Press Ltd All rights reserved 0007-0920/95 $12.00

### Hormone replacement treatment and breast cancer risk: a cooperative Italian study

C La Vecchia[1,2], E Negri[1], S Franceschi[3], A Favero[3], O Nanni[4], R Filiberti[5], E Conti[6], M Montella[7], A Veronesi[8], M Ferraroni[2] and A Decarli[2]

[1]*Istituto di Ricerche Farmacologiche 'Mario Negri', Via Eritrea, 62, 20157 Milan, Italy;* [2]*Istituto di Biometria e Statistica Medica, Università di Milano, Istituto Nazionale Tumori, Via Venezian, 1, 20133 Milan, Italy;* [3]*Centro di Riferimento Oncologico, Via Pedemontana Occ.le, 33081 Aviano (Pordenone), Italy;* [4]*Istituto Oncologico Romagnolo, Ospedale Pierantoni, Via Forlanini, 47100, Forlì, Italy;* [5]*Istituto Nazionale per la Ricerca sul Cancro, Via Benedetto XV, 10, 16132 Genova, Italy;* [6]*Istituto Regina Elena per lo Studio la Cura dei Tumori, Viale Regina Elena, 291, 00100 Rome, Italy;* [7]*Istituto per lo Studio e la Cura dei Tumori Senatore Pascale, Via Cappella dei Cangiani, 80131 Naples, Italy;* [8]*Servizio di Oncologia, Ospedale Civile, Via Vittorio Veneto, 34170 Gorizia, Italy.*

**Summary** The relationship between hormone replacement treatment (HRT) and breast cancer risk was analysed using data from a case-control study conducted between June 1991 and February 1994 in six Italian centres on 2569 patients aged below 75 with histologically confirmed breast cancer and 2588 controls admitted to hospital for a wide spectrum of acute, non-neoplastic, non hormone-related diseases. Ever HRT use was reported by 7.5% of cases and 7.5% of controls, corresponding to a multivariate odds ratio (OR) of 1.2 [95% confidence interval (CI), 0.9-1.5]. The risk increased with increasing duration of use: the ORs were 1.0 for use lasting less than 1 year, 1.3 for 1-4 years and 1.5 for 5 years or more. There was no clear pattern of risk with reference to time since starting use, but the OR was significantly elevated (OR = 2.0, 95% CI 1.3-2.9) for women who had stopped HRT within the last 10 years. No association was observed in those who had stopped HRT more than 10 years ago (OR = 1.0). The increased OR for women who had stopped HRT within the last 10 years was consistent across strata of identified covariates, and was significantly related to duration of use. This study confirms the absence of a strong association between HRT and breast cancer risk, although the risk estimate was above unity for women who had used HRT for 5 years or longer. However, the risk was significantly elevated in the short to medium term after use, particularly for long-term use. This short-term increased risk is consistent with an effect of HRT on one of the later stages of the process of breast carcinogenesis. The flattening of risk with increasing time since stopping, and hence the absence of a long-term cumulative excess in breast cancer risk after stopping HRT exposure, has relevant implications on individual risk assessment and public health.

**Keywords:** breast neoplasms; estrogen replacement therapy; progestational hormones; case-control studies

A large number of epidemiological studies have been published on the possible relationship between hormone replacement treatment (HRT) and breast cancer risk. Although overall the evidence does not support a consistent association between ever use of HRT and subsequent breast cancer risk, several issues remain unsettled (Henderson, 1989; Mack and Ross, 1989; Steinberg *et al.*, 1991; Mann, 1992; Brinton and Schairer, 1993).

First, a few studies conducted on European populations have found elevated relative risks (Hunt *et al.*, 1987; Ewertz, 1988; Bergkvist *et al.*, 1989; La Vecchia *et al.*, 1992). Also, among studies from North America, where the long-term use of oestrogen replacement treatment is more frequent, most investigations have found elevated risks among long-term users (Henderson, 1989; Steinberg *et al.*, 1991; Mann, 1992).

However, no consistent pattern of risk has emerged for other time factors considered. A cohort study found that current use may be particularly relevant (Colditz *et al.*, 1990), thus raising the possibility that HRT may have a late-stage effect on breast carcinogenesis. However, the issue of time-related factors in the HRT-breast cancer question remains open to debate.

The type of preparation is another open question (Colditz *et al.*, 1992): the risk estimates, in fact, tend to be generally higher in European studies (Hunt *et al.*, 1987; Bergkvist *et al.*, 1989; La Vecchia *et al.*, 1992), possibly because of the different types of preparations used. In fact, following heterogeneous prescribing patterns, synthetic oestrogens and oestrogen-progestin combinations tend to be used in Europe, while conjugated oestrogens are more commonly used in North America (Mann, 1992).

Correspondence: C La Vecchia
Received 26 July 1994; revised 7 November 1994; accepted 6 February 1995

Between 1983 and 1990, we conducted a case-control study of HRT and breast cancer risk in the Greater Milan area, Northern Italy, including 3037 cases and 2569 controls (La Vecchia *et al.*, 1986, 1992). Only 5% of cases and 3.5% of controls, however, reported ever use of HRT, corresponding to a relative risk (RR) of 1.3, of borderline significance, and a moderate trend of increased risk with increasing duration of use (RR = 1.5 for longest use).

The prevalence of use of HRT, however, differs in various areas of the country, and has increased over recent years. Thus, to provide updated information on the HRT-breast cancer issue in Italy, we considered data from a cooperative case-control study of breast cancer conducted between 1991 and 1994 in six different areas of the country.

### Subjects and methods

The data were derived from a case-control study of breast cancer, conducted between June 1991 and February 1994 in six Italian areas: Greater Milan, the provinces of Pordenone and Gorizia, the urban area of Genoa, the province of Forlì, in northern Italy, the province of Latina in central Italy, and the urban area of Naples, in southern Italy. The same structured questionnaire and coding manual were used in all study centres, and all interviewers were centrally trained and tested for reliability and reproducibility. On average, less than 4% of cases and controls approached for interview refused to participate.

Cases were women with incident (i.e. diagnosed within the year before interview) histologically confirmed breast cancer, admitted to the major teaching and general hospitals in the areas under surveillance. A total of 2569 cases aged 23-74 years (median age 55 years) were included in the present analysis.

Controls were women residing in the same geographical areas and admitted for acute conditions to the same network of hospitals where cases had been identified. Women were not included if they had been admitted for gynaecological, hormonal or neoplastic diseases. A total of 2588 controls aged 20–74 years (median age 56 years) were interviewed. They were admitted to hospital for a wide spectrum of acute diseases unrelated to known or potential risk factors for breast cancer. Of these, 22% had traumatic conditions (mostly fractures and sprains), 32% non-traumatic orthopaedic disorders (mostly low back pain and disc disorders), 16% were admitted for acute surgical conditions (mostly abdominal, such as acute appendicitis or strangulated hernia), 18% had eye diseases (mostly cataract and retinal detachment) and 12% miscellaneous other illnesses, such as ear, nose and throat and dental disorders.

The structured questionnaire included information on personal characteristics and habits, education and other socioeconomic factors, general lifetime habits, such as smoking, alcohol and coffee consumption, a validated food frequency consumption section, a few indicators of physical activity, gynaecological and obstetric data, related medical history and history of lifetime use of oral contraceptives, hormonal replacement therapies in menopause, and female hormone preparations for other indications, including time and duration of each episode of use and brand name, whenever available. Lists of the most common female hormone preparations (covering over 90% of the market over the last two decades) were provided to assist recall, whenever indicated. All interviews for cases and controls were conducted in hospital.

*Data analysis*

Odds ratios (ORs) of breast cancer, and the corresponding 95% confidence intervals (CIs) for various measures of HRT use were derived using unconditional multiple logistic regression, fitted by the method of maximum likelihood (Baker and Nelder, 1978; Breslow and Day, 1980), including (i) terms for study centre and age in quinquennia only and (ii) terms for study centre, age, education, marital status, family history of breast cancer, history of benign breast disease, parity and age at first birth, age at menarche, type of menopause and age at menopause.

### Results

Table I gives the distribution of breast cancer cases and the comparison group according to age and other major identified covariates. There was no difference for marital status, but cases were more educated, tended to report earlier menarche and later menopause and were less frequently multiparous and in premenopause. They also reported later first birth and more frequently family history of breast cancer and personal history of benign breast diseases. All these factors were considered potential confounders for the HRT–breast cancer analysis, and hence were included in multiple logistic regression equations.

Table II considers various measures of HRT use in the overall dataset. The same proportion of cases and controls (7.5%) reported ever HRT use; thus, the age-adjusted OR was 1.0 and the multivariate OR was 1.2 (95% CI 0.9–1.5). The risk increased with duration of use: the multivariate ORs were 1.0 for use lasting less than 1 year, 1.3 for 1–4 years and 1.5 for 5 years or more. The trend in risk was of borderline significance. There was no clear pattern of risk with time since first HRT use, since the point estimates were 1.2 for use started within 10 years, 1.3 for 10–14 years and 1.1 for 15 years or more. In contrast, when time since last use was considered, the OR was significantly elevated among women who had stopped use within the last 10 years (OR = 2.0, 95% CI 1.3–2.9), but not among those who had been stopped for 10 years or more (OR = 1.0) or among current users (OR = 0.8).

**Table I** Distribution of 2569 cases of breast cancer and 2588 controls[a] according to age, study centre and selected covariates, Italy, 1991–94

| | Cases No. | Cases (%) | Controls No. | Controls (%) |
|---|---|---|---|---|
| Age | | | | |
| <35 | 87 | (3.4) | 140 | (5.4) |
| 35–44 | 383 | (14.9) | 332 | (12.8) |
| 45–54 | 772 | (30.1) | 692 | (26.7) |
| 55–64 | 799 | (31.1) | 804 | (31.1) |
| 65–74 | 528 | (20.6) | 620 | (24.0) |
| Study Centre | | | | |
| Pordenone/Gorizia | 1046 | (40.7) | 1015 | (39.2) |
| Milan | 585 | (22.8) | 623 | (24.1) |
| Genoa | 290 | (11.3) | 310 | (12.0) |
| Forli | 212 | (8.3) | 213 | (8.2) |
| Rome/Latina | 178 | (6.9) | 178 | (6.9) |
| Naples | 258 | (10.0) | 249 | (9.6) |
| Education (years) | | | | |
| <7 | 1259 | (49.0) | 1569 | (60.6) |
| 7–11 | 714 | (27.8) | 642 | (24.8) |
| ≥12 | 582 | (22.7) | 354 | (13.7) |
| Unknown | 14 | (0.5) | 23 | (0.9) |
| Marital status | | | | |
| Never married | 230 | (9.0) | 233 | (9.0) |
| Ever married | 2339 | (91.1) | 2355 | (91.0) |
| Age at menarche (years) | | | | |
| <13 | 1123 | (43.7) | 1068 | (41.3) |
| 13–14 | 1079 | (42.0) | 1098 | (42.4) |
| ≥15 | 363 | (14.1) | 419 | (16.2) |
| Parity | | | | |
| Nulliparous | 402 | (15.7) | 380 | (14.7) |
| 1–2 | 1567 | (61.0) | 1417 | (54.8) |
| ≥3 | 600 | (23.4) | 791 | (30.6) |
| Age at first birth[b] (years) | | | | |
| <25 | 902 | (35.1) | 1179 | (45.6) |
| ≥25 | 1265 | (49.2) | 1029 | (39.8) |
| Menopausal status | | | | |
| Pre/in | 986 | (38.4) | 842 | (32.5) |
| Post | 1578 | (61.4) | 1745 | (67.4) |
| Age at menopause | | | | |
| <50 years | 641 | (25.0) | 843 | (32.6) |
| ≥50 years | 933 | (36.3) | 899 | (34.7) |
| Type of menopause | | | | |
| Natural | 1299 | (50.6) | 1328 | (51.3) |
| Surgical or other | 279 | (10.9) | 410 | (15.8) |
| Family history of breast cancer | | | | |
| No | 2270 | (88.4) | 2453 | (94.8) |
| Yes | 299 | (11.6) | 135 | (5.2) |
| History of benign breast disease | | | | |
| No | 2262 | (88.1) | 2346 | (90.7) |
| Yes | 307 | (11.9) | 242 | (9.3) |

[a]For some variables, the sum of strata does not add up to the total because of missing values. [b]Parous women only.

Selected types of preparation are considered in Table III. The OR for ever *vs* never users was 1.3 for users of conjugated oestrogens only, 0.9 for ever users of other (mainly synthetic) oestrogens and 1.0 for users of other miscellaneous or undefined (including most women with short-term use) HRT. Only ten subjects (six cases and four controls) reported combined oestrogen–progestin therapy, corresponding to an OR of 1.6. There was no significant heterogeneity in the use of various preparations between cases and controls. Absolute numbers were too limited for analyses of the risk of various hormone prescriptions in current users only, or in strata of duration, recency and latency of use.

The pattern of risk for time since last HRT use (<10 and ≥10 years) is further considered in Table IV across strata of selected covariates. The risk estimates were consistently above unity for recent use across strata of age, parity and family history of breast cancer. The association was appar-

**Table II** Distribution of 2569 breast cancer cases and 2588 controls, odds ratios (OR) and 95% confidence intervals (CIs) according to various measures of hormone replacement treatment (HRT) use, Italy, 1991–94

| | Cases | | Controls | | OR (95% CI) | |
|---|---|---|---|---|---|---|
| | No. | (%) | No. | (%) | OR1[a] | OR2[a] |
| HRT use | | | | | | |
| Never | 2376 | (92.5) | 2395 | (92.5) | 1[b] | 1[b] |
| Ever | 193 | ( 7.5) | 193 | ( 7.5) | 1.0 (0.8–1.3) | 1.2 (0.9–1.5) |
| Duration of HRT use (years) | | | | | | |
| <1 | 91 | (3.5) | 98 | (3.8) | 1.0 (0.7–1.3) | 1.0 (0.8–1.4) |
| 1–4 | 73 | (2.8) | 66 | (2.6) | 1.1 (0.8–1.6) | 1.3 (0.9–1.9) |
| ⩾5 | 27 | (1.1) | 23 | (0.9) | 1.3 (0.7–2.2) | 1.5 (0.8–2.6)[c] |
| Unknown | 2 | (0.1) | 6 | (0.2) | – | – |
| Time since first HRT use (years) | | | | | | |
| <10 | 84 | (3.3) | 70 | (2.7) | 1.2 (0.8–1.6) | 1.2 (0.9–1.8) |
| 10–14 | 33 | (1.3) | 29 | (1.1) | 1.2 (0.7–2.0) | 1.3 (0.8–2.2) |
| ⩾15 | 76 | (3.0) | 94 | (3.6) | 0.9 (0.7–1.2) | 1.1 (0.8–1.5) |
| Time since last HRT use (years) | | | | | | |
| Current users | 27 | (1.0) | 32 | (1.2) | 0.8 (0.5–1.4) | 0.8 (0.5–1.4) |
| <10 years | 78 | (3.0) | 44 | (1.7) | 1.8 (1.2–2.6) | 2.0 (1.3–2.9) |
| ⩾10 | 86 | (3.3) | 111 | (4.3) | 0.8 (0.6–1.1) | 1.0 (0.7–1.3) |
| Unknown | 2 | (0.1) | 6 | (0.2) | – | – |

[a]Estimates from unconditional multiple logistic regression models including term for age, study centre (OR1), plus marital status, education, body mass index, age at menarche, nulliparity, age at first birth, menopausal status, age at menopause, type of menopause, history of benign breast disease and family history of breast cancer (OR2). [b]Reference category. [c]$\chi_1^2$, trend = 3.62, $P \cong 0.05$.

**Table III** Odds ratios[a] (ORs) and 95% confidence intervals (CIs) of breast cancer according to ever use of various preparations of hormone replacement treatment, Italy, 1991–94

| Type of preparation | Cases | Controls | OR (95% CI) |
|---|---|---|---|
| Never users | 2376 | 2395 | 1[b] |
| Conjugated oestrogens | 30 | 27 | 1.3 (0.8–1.6) |
| Other oestrogens only | 32 | 41 | 0.9 (0.7–1.4) |
| Oestrogens and progestins | 6 | 4 | 1.6 (0.4–6.3) |
| Miscellaneous, other or undefined | 125 | 121 | 1.0 (0.8–1.5) |

[a]Estimates from unconditional multiple logistic regression models, including terms for age, study centre, education, body mass index, parity, age at menopause and family history of breast cancer. [b]Reference category.

ently stronger in less educated women, in those of higher body mass index and in those reporting later first birth and menopause, but not history of benign breast disease. None of the interaction terms, however, was significant. Likewise, there was no evidence of interaction with alcohol drinking, any other dietary factor considered–including a measure of total fat and total calorie intake–and cigarette smoking (data not shown). There was no evidence of association between HRT use stopped for 10 or more years and breast cancer risk.

Table V considers the interaction between time since last use and duration of HRT use. For women who had stopped using HRT within the last 10 years, the ORs were 1.4 for use of less than 5 years and 1.7 for use lasting 5 years or more, and the trend in risk was of borderline significance. In contrast, there was no pattern of risk with duration of HRT use for women who had stopped for 10 years or more.

**Table IV** Odds ratios[a] of breast cancer according to time since last use of hormone replacement treatment as compared with never users in strata of selected covariates, Italy, 1991–94

| Strata | Time since last use (years) <10 | ⩾10 |
|---|---|---|
| Age (years) | | |
| 50–59 | 1.4 | 0.6 |
| 60–79 | 2.8[b] | 1.2 |
| Education (years) | | |
| <7 | 2.1[b] | 0.9 |
| ⩾7 | 1.0 | 1.0 |
| Body mass index (kg m$^{-2}$) | | |
| <25 | 1.3 | 1.1 |
| ⩾25 | 1.9[b] | 0.8 |
| Age at menarche (years) | | |
| <13 | 1.8[b] | 0.9 |
| ⩾13 | 2.0[b] | 1.0 |
| Parity | | |
| Nulliparous | 1.5 | 0.8 |
| 1–2 | 1.4 | 1.0 |
| ⩾3 | 1.5 | 1.0 |
| Age at first birth (years) | | |
| <25 | 1.2 | 1.2 |
| ⩾25 | 1.9[b] | 1.0 |
| Family history of breast cancer | | |
| No | 1.5 | 1.0 |
| Yes | 3.7 | 0.9 |
| History of benign breast disease | | |
| No | 1.7[b] | 0.9 |
| Yes | 1.0 | 0.8 |
| Type of menopause | | |
| Natural | 1.8[b] | 1.0 |
| Surgical or other | 2.6[b] | 0.9 |
| Age at menopause (years) | | |
| <50 | 1.6 | 0.9 |
| ⩾50 | 2.4[b] | 1.4 |

[a]Estimates from unconditional multiple logistic regression models including terms for age, study centre, education, body mass index, parity, age at menopause and family history of breast cancer. [b]$P < 0.05$.

### Discussion

The present study confirms the absence of strong association between menopausal replacement treatment and breast cancer risk, although the risk estimate was above unity in women who had used HRT for 5 years or longer. In a sense, its major value and originality derives from the study population. In fact, this is one of the few available European studies, and several investigations based on European populations (Hunt *et al.*, 1987; Ewertz 1989; Bergkvist *et al.*, 1989), including a previous Italian one (La Vecchia *et al.*, 1992), tended to support the existence of an association between HRT and breast cancer risk, which could be explained in terms either of selection of menopausal replacement

**Table V** Odds ratios[a] of breast cancer according to duration of hormone replacement treatment (HRT) and time since last use as compared with never users, Italy, 1991–1994

| | *Time since last HRT use (years)* | |
|---|---|---|
| *Duration (years)* | *<10* | *≥10* |
| <5 | 1.4 (1.0–2.0) | 1.0 (0.7–1.3) |
| ≥5 | 1.7 (0.8–3.7)[b] | 1.2 (0.5–2.9) |

[a]Estimates from an unconditional logistic regression model including terms for age, study centre, marital status, education, body mass index, age at menarche, nulliparity, age at first birth, menopausal status, age at menopause, type of menopause, history of benign breast disease and family history of breast cancer. [b]$\chi_1^2$, trend = 3.71, $P \cong 0.05$.

treatment users or of different compositions of preparations used in Europe (with a higher prevalence of synthetic oestrogens and oestrogen–progestin preparations in Europe) and North America, or both.

However, there was an elevated risk for women who had recently stopped HRT use, and a trend with duration which was restricted to this subgroup. This is consistent with the elevated risk observed in several studies, largely based on recent long-term use (Henderson, 1989; Steinberg *et al.*, 1991). A cohort study, in particular, found that current use was specifically related to breast cancer risk (Colditz *et al.*, 1990).

This short-term increased risk is also reflected in the pattern of breast cancer risk observed after a full-time pregnancy or suggested after stopping oral contraceptive use, with a short-term elevated risk that tends to level off or reverse after 5–10 years (Bruzzi *et al.*, 1988; La Vecchia *et al.*, 1990). This short-term effect is consistent across strata of major identified covariates. In terms of the multistage model of carcinogenesis, this would imply that HRT has a late-stage effect on breast carcinogenesis (Day and Brown, 1980), as on other female hormone-related neoplasms, such as endometrial cancer (La Vecchia *et al.*, 1984). In terms of public health implications, these results suggest that the elevated breast cancer risk is restricted to the short period after stopping use, in the absence of a long-term, and hence a cumulative, excess risk after stopping HRT use.

In this study, however, there was no excess risk for current users. If not due to chance, this may be attributable to the shorter duration in current users (who had not yet completed their period of use) or to some selection mechanisms, which may lead women at high risk of breast cancer to withdraw from HRT use.

Although there was no significant interaction between exogenous oestrogen use and age at diagnosis, there was some suggestion of higher risk in the elderly, which is consistent with the biological decline in endogenous hormones with age (Cauley *et al.*, 1989; Brinton and Schairer, 1993), as well as with data from other case–control studies (Brinton *et al.*, 1986; Wingo *et al.*, 1987; Palmer *et al.*, 1991; Kaufman *et al.*, 1991). Along a similar line of reasoning (Brinton and Schairer, 1993), the OR was somewhat (though not significantly) higher in women with surgical menopause than in those with natural menopause. No meaningful interactions or subgroup effect were observed with any of the other variables considered, such as family history of breast cancer, body mass index or alcohol drinking, which have been debated in the past (Brinton and Schairer, 1993).

Most of the limitations and strengths of this study are common to other hospital-based case–control studies (Mantel and Haenszel, 1959). Thus, although this study was not population based, cases were identified in the major teaching and general hospitals of the areas under surveillance, limiting the possibility of selection bias. Still, some selection bias may be related to the definition of the comparison group. For this reason, only acute conditions, unrelated to known or potential risk factors for breast cancer, or to correlates of HRT use in this population (Parazzini *et al.*, 1993), were included in the comparison group. Further, the hospital-based design may improve the comparability of drug recall by cases and controls, and – of specific interest to this study – the participation of cases and controls was practically complete. The potential confounding effect of several covariates was allowed for in the analysis, but did not have an appreciable impact on any of the relative risk estimates. Indeed, the multivariate relative risk estimates were systematically higher than the age-adjusted ones, suggesting that unadjusted values were somewhat, although moderately, underestimated. Of greater concern, in the interpretation of this study, was the low prevalence of menopausal replacement treatment in this Italian population, which not only hampered detailed analysis of subgroups and interactions, but might have concealed some residual selection mechanisms.

The small absolute numbers, particularly of users of oestrogen–progestin combinations, also precluded any meaningful inference on different types of preparations. Still, there was little indication in this dataset of association with any specific type of preparation, or combination of treatments.

These limitations and potential problems notwithstanding, it is unlikely that any selection, information or confounding bias would have led to systematic and substantial underestimation of the association between HRT and breast cancer risk. Thus, these data help to better assess the pattern of breast cancer risk for various time-related aspects of HRT use in a southern European population.

In more general terms of risk assessment and implications for prescription, these data indicate that there is a moderate increase in breast cancer in the short to medium term after use, but are largely reassuring for the ultimate long-term impact of HRT on breast carcinogenesis.

**Acknowledgements**

This work was conducted within the framework of the CNR (Italian National Research Council)'Risk factors for Disease' (Contract No. 94.00695.PF41), Clinical Applications of Oncological Research (Contracts No. 94.01321.PF39, 94.01119.PF39 and 94.1268.P539) and with contributions from the Italian Association for Cancer Research and the Italian League against Tumours, Milan. The authors wish to thank Dr Fabio Barbone, University of Udine, for useful comments, Mrs Judy Baggott, Ms M Paola Bonifacino and the G.A. Pfeiffer Memorial Library staff for editorial assistance.

**References**

BAKER RJ AND NELDER JA. (1978). *The GLIM System*, Release 3. Numerical Algorithms Group: Oxford.

BERGKVIST L, ADAMI HO, PERSSON I, HOOVER R AND SCHAIRER C. (1989). The risk of breast cancer after estrogen and estrogen–progestin replacement. *N. Engl. J. Med.*, **321**, 293–297.

BRESLOW NE AND DAY NE. (1980). *Statistical Methods in Cancer Research*, Vol. 1, *The Analysis of Case–Control Studies*. IARC Scientific Publication No. 32. IARC: Lyon.

BRINTON LA AND SCHAIRER C. (1993). Estrogen replacement therapy and breast cancer risk. *Epidemiol. Rev.*, **15**, 66–79.

BRINTON LA, HOOVER R AND FRAUMENI JF JR. (1986). Menopausal oestrogens and breast cancer risk: an expanded case–control study. *Br. J. Cancer*, **54**, 825–832.

BRUZZI P, NEGRI E, LA VECCHIA C, DECARLI A, PALLI D, PARAZZINI F AND ROSSELLI DEL TURCO M. (1988). Short term increase in risk of breast cancer after full term pregnancy. *Br. Med. J.*, **297**, 1096–1098.

CAULEY JA, GUTAL JP, KULLER LH, LEDONNE D AND POWELL JG. (1989). The epidemiology of serum sex hormones in postmenopausal women. *Am. J. Epidemiol.*, **129**, 1120–1131.

COLDITZ GA, STAMPFER MJ, WILLETT WC, HENNEKENS CH, ROSNER B AND SPEIZER FE. (1990). Prospective study of estrogen replacement therapy and risk of breast cancer in postmenopausal women. *JAMA*, **264**, 2648–2653.

COLDITZ GA, STAMPFER MJ, WILLETT WC, HUNTER DJ, MANSON JAE, HENNEKENS CH, ROSNER BA AND SPEIZER FE. (1992). Type of postmenopausal hormone use and risk of breast cancer: 12-year follow-up from the Nurses' Health Study. *Cancer Causes Control*, **3**, 433-439.

DAY NE AND BROWN CC. (1980). Multistage models and primary prevention of cancer. *J. Natl. Cancer Inst.*, **64**, 977-989.

EWERTZ M. (1988). Influence of non-contraceptive exogenous and endogenous sex hormones on breast cancer risk in Denmark. *Int. J. Cancer*, **42**, 832-838.

HENDERSON BE. (1989). The cancer question: an overview of recent epidemiologic and retrospective data. *Am. J. Obstet. Gynecol.*, **161**, 1859-1864.

HUNT K, VESSEY M, McPHERSON K AND COLEMAN M. (1987). Long-term surveillance of mortality and cancer incidence in women receiving hormone replacement therapy. *Br. J. Obstet. Gynaecol.*, **94**, 620-635.

KAUFMAN DW, PALAMER JR, DE MOUZON J, ROSENBERG L, STOLLEY PD, WARSHAUER ME, ZAUBER AG AND SHAPIRO S. (1991). Estrogen replacement therapy and the risk of breast cancer: results from the case-control surveillance study. *Am. J. Epidemiol.*, **134**, 1375-1385.

LA VECCHIA C, FRANCESCHI S, DECARLI A, GALLUS G AND TOGNONI G. (1984). Risk factors for endometrial cancer at different ages. *J. Natl Cancer Inst.*, **73**, 667-671.

LA VECCHIA C, DECARLI A, PARAZZINI F, GENTILE A, LIBERATI C AND FRANCESCHI S. (1986). Non-contraceptive oestrogens and the risk of breast cancer in women. *Int. J. Cancer*, **38**, 853-858.

LA VECCHIA C, BRUZZI P AND BOYLE P. (1990). Some further consideration on the role of oral contraceptives in breast carcinogenesis. *Tumori*, **76**, 220-224.

LA VECCHIA C, NEGRI E, FRANCESCHI S AND PARAZZINI F. (1992). Non-contraceptive oestrogens and breast cancer: an update (letter). *Int. J. Cancer*, **50**, 161-162.

MACK TM AND ROSS RK. (1989). Risks and benefits of long-term treatment with estrogens. *Schweiz. Med. Wochenschr.*, **119**, 1811-1820.

MANN RD (ed.) (1992). *Hormone Replacement Therapy and Breast Cancer Risk*. Parthenon: Park Ridge, NJ.

MANTEL N AND HAENSZEL W. (1959). Statistical aspects of the analysis of data from retrospective studies of disease. *J. Natl Cancer Inst.*, **22**, 719-748.

PALMER JR, ROSENBERG L, CLARKE EA, MILLER DR AND SHAPIRO S. (1991). Breast cancer risk after estrogen replacement therapy: results from the Toronto Breast Cancer Study. *Am. J. Epidemiol.*, **134**, 1386-1395.

PARAZZINI F, LA VECCHIA C, NEGRI E, BIANCHI C AND FEDELE L. (1993). Determinants of estrogen replacement therapy use in Northern Italy. *Rev. Epidemiol. Sante Publique*, **41**, 53-58.

STEINBERG KK, THACKER SB, SMITH SJ, STROUP DF, ZACK MM, FLANDERS WD AND BERKELMAN RL. (1991). A meta-analysis of the effect of estrogen replacement therapy on the risk of breast cancer. *JAMA*, **265**, 1985-1990.

WINGO PA, LAYDE PM, LEE NC, RUBIN G AND HOWARD WO. (1987). The risk of breast cancer in postmenopausal women who have used estrogen replacement therapy. *JAMA*, **257**, 209-215.